# CRISPR1-mediated virulence and erythromycin resistance stabilization drives regional dominance of *Streptococcus agalactiae* ST10 in China

**DOI:** 10.3389/fcimb.2026.1703530

**Published:** 2026-03-06

**Authors:** Xiaohui Qiao, Ke Dai, Jilong Su, Yimeng Zhang, Yuxi Bai, Xinhua Zhang, Li Zhang

**Affiliations:** 1 Department of Pediatrics, Shanxi Medical University, Taiyuan, China; 2 Xiangyuan County Center for Disease Control and Prevention, Changzhi, Shanxi, China; 3 Department of Neonatal, Shanxi Children’s Hospital (Shanxi Maternal and Child Health Hospital), Taiyuan, China; 4 Children's Hospital of Shanxi Medical University, Taiyuan, China; 5 Department of Newborn Disease Screening Center, Shanxi Children’s Hospital (Shanxi Maternal and Child Health Hospital), Taiyuan, China

**Keywords:** *Streptococcus agalactiae*, CRISPR1, *ermB*, molecular epidemiology, ST10, virulence genes

## Abstract

**Introduction:**

*Streptococcus agalactiae* (Group B Streptococcus, GBS) is a major pathogen of neonatal infections worldwide, with significant geographical variation in its prevalent sequence types (STs). In Shanxi, China, ST10 has emerged as the dominant lineage in perinatal infections, yet the mechanisms underlying its regional dominance remain unclear. This study aimed to investigate the molecular epidemiological basis for the predominance of ST10 in this region.

**Methods:**

We analyzed 55 clinical *Streptococcus agalactiae* isolates (21 invasive, 34 colonizing) collected from a hospital in Shanxi, China. Multilocus sequence typing (MLST) was performed to determine sequence types. Virulence genes and the CRISPR1 (clustered regularly interspaced short palindromic repeats, CRISPR) system were detected by polymerase chain reaction (PCR). Statistical analyses were conducted to assess associations between ST10, CRISPR1, and virulence gene carriage.

**Results:**

ST10 accounted for 50.9% (28/55) of all isolates, predominating in both invasive (13/21, 61.9%) and colonizing (15/34, 44.1%) groups. Invasive ST10 strains universally carried the virulence genes *cylE*, *hylB*, *scpB*, and *bca* (100%) and exhibited complete erythromycin resistance mediated by *ermB* (100%). The CRISPR1 system was highly prevalent in ST10 isolates (26/28, 92.9%), and its presence was significantly associated with the virulence genes *cylE* (69.8% vs 25.0%; P=0.014) and *hylB* (62.8% vs 16.7%; P=0.012).

**Discussion:**

The findings delineate a "high-virulence/controlled-resistance" phenotype in the locally dominant ST10, characterized by a conserved virulence gene, stable chromosomal *ermB* resistance, and a high prevalence of CRISPR1. This suggests CRISPR1 may contribute to the fitness of the ST10 lineage by stabilizing chromosomal virulence and resistance determinants while potentially restricting plasmid acquisition. This study establishes CRISPR1 as a potential key driver of ST10 adaptation and a possible predictive marker for monitoring high-risk clones, providing a rationale for regionally tailored prophylaxis strategies.

## 1 Introduction

*Streptococcus agalactiae* (Group B Streptococcus, GBS) is a leading cause of neonatal invasive disease globally, in severe cases, it can cause sepsis and meningitis, resulting in significant mortality (Sinha et al., 2016; Gonçalves et al., 2022; Dangor et al., 2023). The pathogenicity of GBS is mediated by a repertoire of conserved virulence, including cytolysin, hyaluronidase, and so on, which collectively facilitate epithelial colonization, immune evasion, and systemic dissemination (Hernandez et al., 2022; Liu and Liu, 2022). Molecular epidemiological surveillance has revealed considerable geographical difference in the predominant GBS sequence types (STs). While ST17 is frequently associated with neonatal meningitis in Western (Jones et al., 2003; Deshayes de Cambronne et al., 2021), distinct lineages appear to dominate in other regions, underscoring the influence of local evolutionary and ecological pressures on strain success.

In northern China, multi-center studies of perinatal isolates have identified ST10 as the predominant lineage (Zhang et al., 2021b; Bai et al., 2022). Our previous study linked the presence of the clustered regularly interspaced short palindromic repeats (CRISPR) systems in ST10 to the macrolide resistance gene *ermB* (Zhang et al., 2021b; Bai et al., 2022), suggesting a potential adaptive role for CRISPR in maintaining resistant clones. The ecological success of dominant lineages often hinges on the stable inheritance and adaptive evolution of key genetic determinants. In GBS, macrolide resistance can be mediated by either chromosomal *ermB*, which spreads vertically, or by plasmid-borne *ermA*, spreading horizontally via mobile genetic elements (de Azavedo et al., 2001; Puopolo et al., 2007; Woodbury et al., 2008; Campelo et al., 2012; Kamińska et al., 2024). Evidence from other species, like *Enterococcus faecalis*, shows CRISPR-Cas systems can modulate this dynamic by blocking plasmid uptake (Sun et al., 2019; Evans et al., 2020), while preserving chromosomal resistance genes (Hullahalli et al., 2017; Gholizadeh et al., 2021). Nevertheless, the specific function of CRISPR systems in shaping virulence and clonal adaptation in GBS remains unclear.

Several knowledge gaps persist in the current literature. First, while the predominance of ST10 in certain regions has been documented (Cruz-López et al., 2021; Zhang et al., 2021a), the molecular mechanisms underpinning its epidemiological success are not fully elucidated. Second, although CRISPR systems have been implicated in bacterial adaptation (Zhao et al., 2018; Cruz-López et al., 2021), their specific impact on virulence gene maintenance in GBS has not been mechanistically explored.

To address these gaps, the study was designed to investigate the dominant sequence type and the molecular basis for its prevalence among clinical isolates from Shanxi, China. Based on prior observations, two hypotheses were proposed: (i) CRISPR1-mediated exclusion of mobile genetic elements relieves the fitness burden of plasmid maintenance, thereby positively selecting for and stabilizing chromosomal virulence determinants (Zhang et al., 2021a; Bai et al., 2022); (ii) Phage defense activity protects the genome from invasive recombination, preserving the integrity and function of key adaptive clusters (Le Rhun et al., 2019; Fillol-Salom et al., 2022). To test these hypotheses, a molecular profiling was employed, including multilocus sequence typing (MLST), virulence gene detection by polymerase chain reaction (PCR), and CRISPR1 system screening. This work aims to provide mechanistic insights into how CRISPR systems may co-optimize virulence and resistance traits during clonal expansion in high-risk populations.

## 2 Materials and methods

### 2.1 Strain collection and ethical compliance

We retrospectively analyzed 55 clinical *Streptococcus agalactiae* isolates collected at Shanxi Medical University Children’s Hospital between January 2017 and January 2024. The isolates originated from two distinct cohorts: (1) vaginal swabs of colonized pregnant women (≥35 gestational weeks), with the inclusion criterion being a single-positive culture in 37°C, 5% CO_2_, Todd-Hewitt broth enrichment; and (2) blood or cerebrospinal fluid (CSF) cultures from neonates with invasive GBS disease (onset within 72 hours post-delivery), confirmed by culture positivity alongside clinical symptoms of sepsis or meningitis. Invasive GBS disease including sepsis, meningitis, pneumonia, or focal infections, was defined according to standardized clinical and microbiological criteria. Cases were classified as early-onset disease (EOD, 0–7 days), late-onset disease (LOD, 8–90 days), or late-late-onset disease (LLOD, occurring after 90 days of age).

The study protocol (Approval No. IRB-KYYN-2021-001(16)) adhered to the Declaration of Helsinki, with waived informed consent for anonymized residual clinical specimens.

### 2.2 Multilocus sequence typing

The method of Jones et al. (2003) was followed (Jones et al., 2003), selecting seven conserved housekeeping genes (including *adhP, atr, gdh, glcK, pheS, sdhA, and tkt*) for PCR amplification and sequencing. The allele number, STs was conducted using the MLST database (http://pubmlst.org/sagalactiae/).

### 2.3 Detection of virulence genes

Using conventional PCR under conditions similar to MLST. We targeted virulence-associated genes involved in key pathogenic processes of GBS, including adhesion, invasion, immune evasion, and tissue dissemination. Specifically, β-hemolysin (*cylE*) and hyaluronidase (*hylB*) were selected for their respective roles in enhancing host cell invasion and promoting bacterial dissemination and tissue invasiveness (Oviedo et al., 2013; Mudzana et al., 2021; Verma et al., 2023). The laminin-binding protein gene (*lmb*) and the α and β antigens of the C protein (*bca* and *bac*) were included for their role in mediating bacterial adhesion to epithelial cells (Oviedo et al., 2013; Jiang et al., 2016; Mudzana et al., 2021). Additionally, C5a peptidase (*scpB*) impedes neutrophil recruitment, and the Rib surface protein gene (*rib*) contributes to immune evasion through expression of a protease-resistant surface protein (Lindahl et al., 2005; Mudzana et al., 2021). PCR amplification products were separated by electrophoresis on a 1.5% agarose gel, with D2000 DNA Marker used as a molecular weight reference.

### 2.4 CRISPR system analysis

PCR was used to detect the presence of the CRISPR system in GBS strains (Beauruelle et al., 2017). Specific primers(oligonucleotide pairs CRISPR1-PCRF/CRISPR1-PCRR) were used for amplification, then PCR products were sequenced by using internal sequencing primers CRISPR-SEQF/CRISPR- SEQR. CRISPR sequences were analyzed by using the CRISPRs web server (https://crispr.i2bc.paris-saclay.fr/).

### 2.5 Data statistics

All experimental data were entered into IBM SPSS Statistics 26 for statistical analysis. Technical data were described by frequency and percentage, and analyzed by χ² test or Fisher’s exact probability method. A P-value of less than 0.05 was considered statistically significant.

## 3 Results

### 3.1 Isolate collection and serotyping

**Figure 1:**
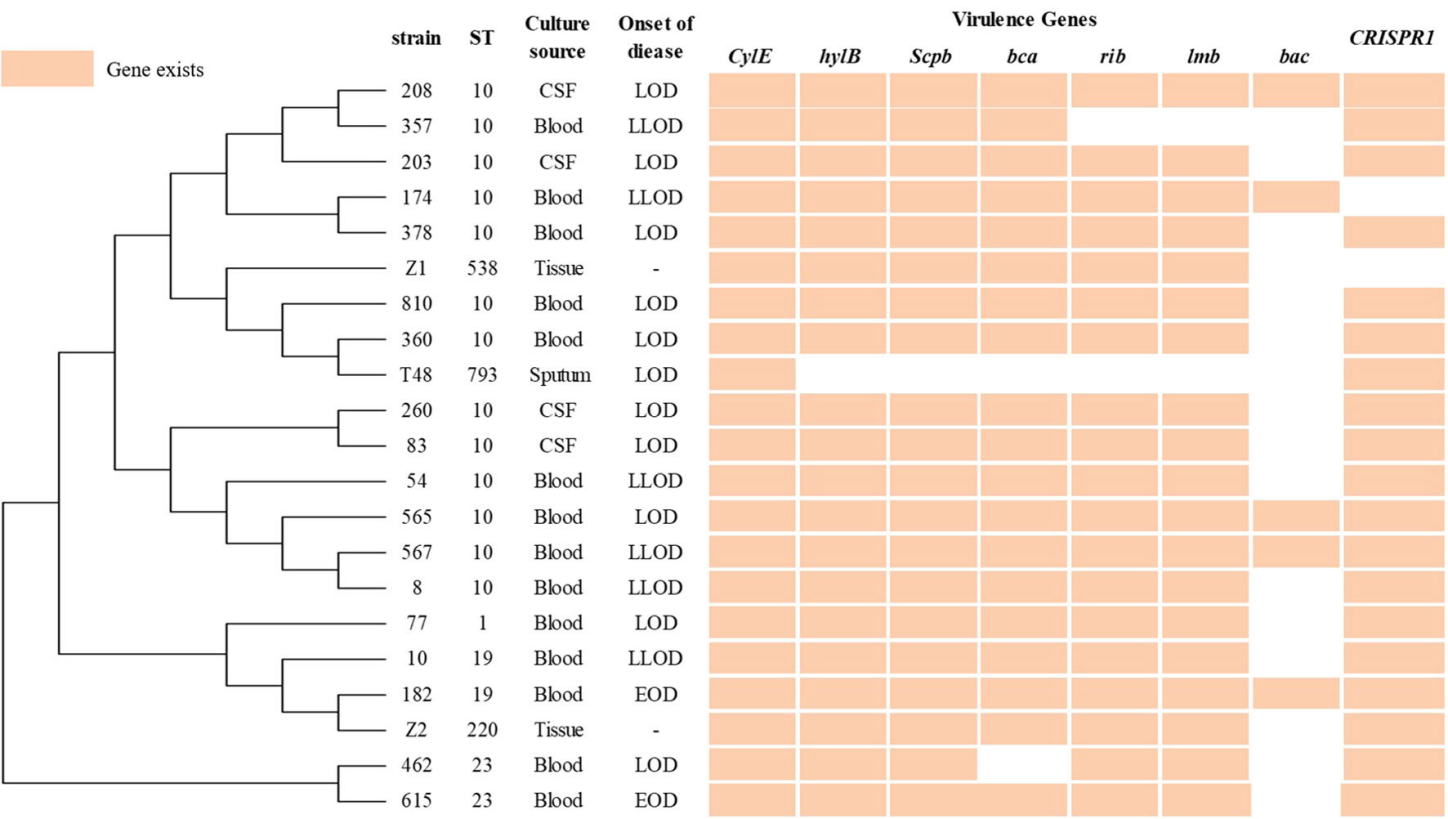
Phylogeny constructed from the multi-locus sequence typing (MLST) profiles of 7 housekeeping genes of 21 invasive GBS.

In total, 55 GBS strains were collected, among which 34 colonizing strains were obtained from vaginal swabs of pregnant women. The remaining 21 were invasive strains, blood (14/21, 70.2%) accounted for the main specimens, followed by CSF (n=4), tissue (n=2), and sputum (n=1). The invasive GBS isolates corresponded to 2 cases of EOD, 11 LOD, 6 LLOD, and 2 placental infection tissues from pregnant women who experienced abortion. **Figure 1** provides detailed information on the invasive isolates (**Figure 1**). A total of 11 distinct STs were detected, with ST10 (28/55, 50.91%) being the most prevalent, predominating in both invasive (13/21) and colonizing strains (15/34). The second most common ST was ST19, accounting for 16.36%. The following sequence types included ST1 (9.09%), ST23 (5.45%), etc. (**Table 1**).

**Table 1:** Characteristics of *Streptococcus agalactiae* isolates by sequence type (ST).

MLST	GBS strains(n/%)	Invasive strains(n/%)	Colonizing strains(n/%)
ST10	28/50.91	13/61.90	15/44.12
ST19	9/16.36	2/9.52	7/20.59
ST1	5/9.09	1/4.76	4/11.76
ST23	3/5.45	2/9.52	1/2.94
ST27	2/3.64	0	2/5.88
ST12	2/3.64	0	2/5.88
ST17	2/3.64	0	2/5.88
ST24	1/1.82	0	1/2.94
ST538	1/1.82	1/4.76	0
ST220	1/1.82	1/4.76	0
ST793	1/1.82	1/4.76	0
Total	55/100	21/100	34/100

### 3.2 Virulence gene distribution in ST10 versus non-ST10 GBS

Analysis of virulence gene distribution among 55 GBS isolates revealed distinct patterns between ST10 and non-ST10 strains (**Table 2**; **Figure 1**). Among the 21 invasive isolates, all ST10 strains (n=13) carried the *cylE, hylB, scpB*, and *bca* genes (100%). Although a trend toward higher positivity in ST10 isolates for certain genes (e.g., *bca*, 100% *vs*. 75.0%), none of these reached statistical significance. Within the 34 non-invasive isolates, ST10 strains exhibited significantly higher carriage rates for *scpB*, *bca*, and *bac* (all P<0.001) compared to non-ST10 strains, whereas the gene *rib* was more frequently detected in non-ST10 isolates (57.9% *vs*. 13.3%, P = 0.013).

**Table 2:** Comparative analysis of virulence gene carriage rates among strain groups.

Virulence Gene	Invasive ST10	Invasive non-ST10	χ²	P-value	Non-invasive ST10	Non-invasive non-ST10	χ²	P-value
	n=13	n=8			n=15	n=19		
*cylE*	100% (13/13)	100% (8/8)		1.000	46.7% (7/15)	26.3% (5/19)	1.520	0.288
*hylB*	100% (13/13)	87.5% (7/8)	1.706	0.191	40.0% (6/15)	15.8% (3/19)	2.524	0.139
*scpB*	100% (13/13)	87.5% (7/8)	1.706	0.191	100% (15/15)	42.1% (8/19)	12.838	<0.001
*bca*	100% (13/13)	75.0% (6/8)	3.592	0.058	60.0% (9/15)	5.3% (1/19)	12.097	<0.001
*rib*	92.3% (12/13)	87.5% (7/8)	0.133	0.761	13.3% (2/15)	57.9% (11/19)	7.048	0.013
*lmb*	92.3% (12/13)	87.5% (7/8)	0.133	0.761	100% (15/15)	89.5% (17/19)	1.678	0.492
*Bac*	30.8% (4/13)	12.5% (1/8)	0.911	0.340	80.0% (12/15)	5.3% (1/19)	19.826	<0.001

### 3.3 Distribution of CRISPR1 system across different STs

Among the 55 GBS strains analyzed, 43 were identified as CRISPR1-positive, indicating a carriage rate of 78.2%. The distribution of the CRISPR1 system displayed specific patterns by STs (**Table 3**; **Figure 1**), with ST10 strains showing a high prevalence in CRISPR1 system (26/28, 92.9%), while ST19 exhibited a lower frequency (4/9, 44.4%). Statistical significance was indicated for these two STs (P < 0.05). The remaining STs were represented by smaller numbers of isolates, and no statistically significant differences in CRISPR1 distribution were detected.

**Table 3:** Distribution of the CRISPR1 system among *Streptococcus agalactiae* STs.

Sequence Type (ST)	CRISPR1+	CRISPR1-	Total	χ²	P-value
	n=43	n=12	n=55		
ST10	26	2	28	5.555	0.018
ST19	4	5	9	5.010	0.025
ST1	2	3	5	2.561	0.110
ST23	3	0	3	0.049	0.824
ST12	1	1	2	0.012	0.912
ST17	2	0	2	0.000	1.000
ST27	2	0	2	0.000	1.000
ST24	1	0	1	0.000	1.000
ST220	1	0	1	0.000	1.000
ST538	0	1	1	0.474	0.491
ST793	1	0	1	0.000	1.000

### 3.4 Association between CRISPR1 and carriage of virulence genes

**Figure 2:**
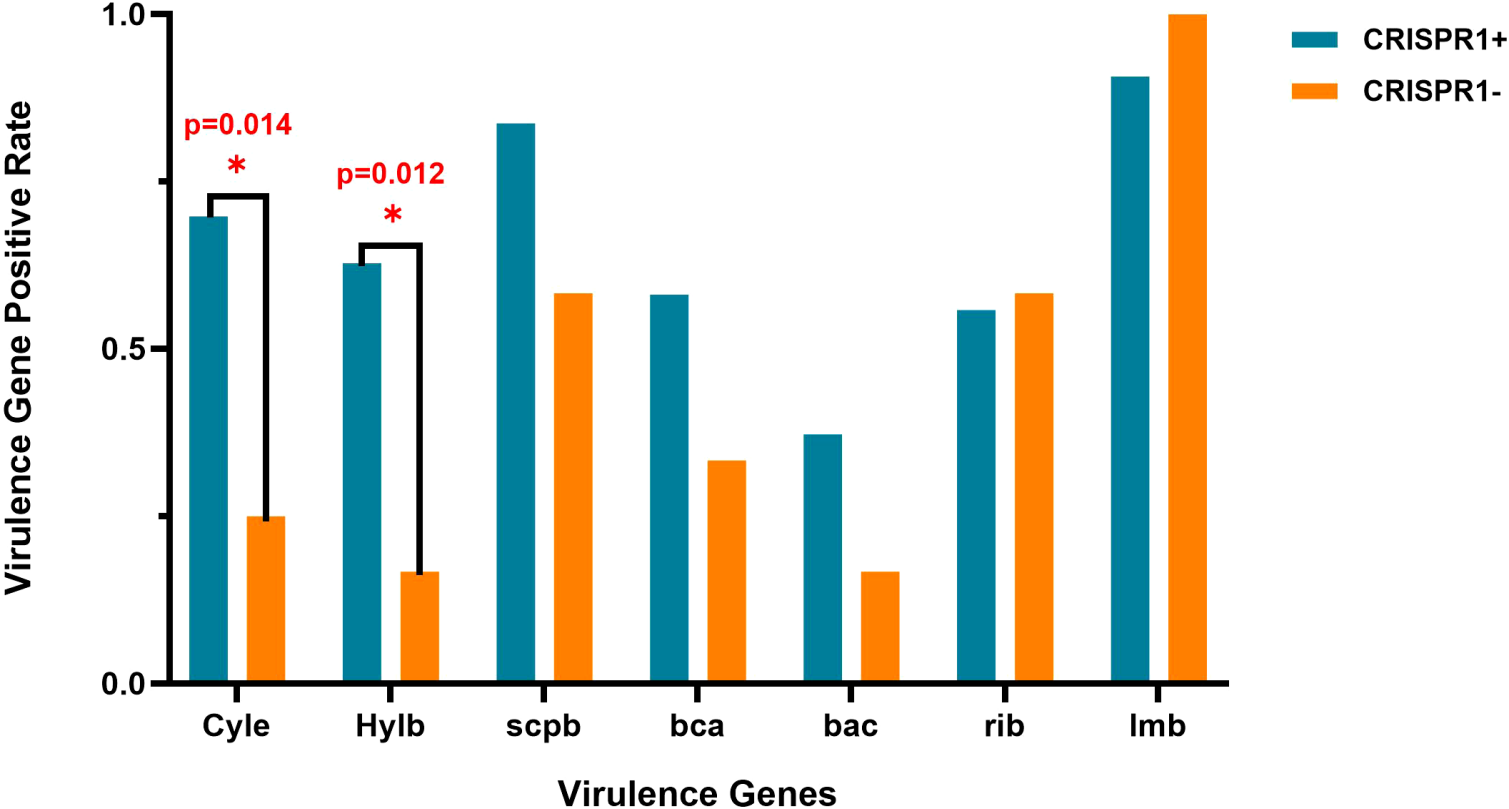
Association between CRISPR1 system and virulence genes in *Streptococcus agalactiae* isolates, the asterisk (*) denotes a statistically significant difference between groups (P < 0.05), as determined by Fisher’s discriminant analysis.

A significant association was found between the presence of CRISPR1 and the carriage of the virulence genes cylE (69.8% *vs* 25.0%; P = 0.014) and hylB (62.8% *vs* 16.7%; P = 0.012), which were more frequently identified in CRISPR1-positive isolates (**Figure 2**). No significant associations were observed for other virulence genes (scpB, bca, bac, rib, lmb; all P > 0.05).

## 4 Discussion

This study delineates the regional epidemiological dominance of Streptococcus agalactiae ST10 in perinatal settings in Shanxi, China. The core findings indicate that ST10 predominates among clinical isolates and is characterized by a conserved virulence gene profile, with a significant prevalence of the CRISPR1 system. These observations align with emerging evidence on CRISPR systems’ multifunctional roles in bacterial adaptation while highlighting region-specific evolutionary trajectories in GBS pathogenesis.

The ST10 lineage accounted for 50.9% (28/55) of isolates in our cohort, dominating both invasive (13/21, 61.9%) and colonizing (15/34, 44.1%) populations. This pattern aligns with reports from northern China (Liu and Liu, 2022; Zhou et al., 2024; Yu et al., 2026) but contrasts with the predominance of ST19 documented in most other parts of the country (Li et al., 2023; Chen et al., 2024), highlighting significant inter-provincial variation. Moreover, the ST17 (frequently reported in Western and associated with neonatal invasive infections (Gonçalves et al., 2022)) was low in our study, which is attributable to the specific clinical sources of the isolates. These data supplement the regional epidemiological map of GBS in China and help clarify the local clonal dynamics, which is crucial for informing regionally tailored prevention strategies. Notably, ST10 strains universally retained four virulence genes (cylE, hylB, scpB, and bca), contrasting with variable carriage in non-invasive strains (**Table 2**). This aligns with established mechanisms of GBS neuroinvasion where surface proteins facilitate central nervous system penetration (Doran et al., 2005), suggesting ST10’s potential neurotropic characteristics. The complete virulence in invasive ST10 isolates may explain its enhanced pathogenic potential compared to colonizing-dominant ST19 strains (13.2% prevalence), consistent with emerging evidence linking specific virulence gene combinations to tissue tropism in GBS (Jones et al., 2003).

CRISPR-Cas systems have been increasingly recognized for their role beyond phage defense, including in modulating horizontal gene transfer and stabilizing genomic islands in various bacterial species (Hullahalli et al., 2017; Zhao et al., 2018; Sun et al., 2019; Cruz-López et al., 2021). In GBS, however, direct evidence linking CRISPR systems to adaptive traits has been limited. The high prevalence of CRISPR1 (92.9%) within the dominant ST10 lineage, coupled with its significant statistical association with the retention of key virulence genes (cylE, hylB), provides correlative evidence supporting a potential stabilizing function. This observation resonates with findings in *Streptococcus pyogenes*, where CRISPR arrays have been implicated in maintaining virulence genes by preventing excision via mobile elements (Nozawa et al., 2011; Firestone et al., 2025). We thus propose that in ST10 strains, CRISPR1 likely safeguards the virulence gene cluster (cylE-hylB-scpB-bca) through two mechanisms: direct integration of spacers targeting phage-derived sequences flanking the virulence loci, and transcriptional coupling of the CRISPR-Cas operon with adjacent pathogenicity islands, enabling their co expression.

Furthermore, integrating antimicrobial resistance patterns into this model enhanced its predictive performance. Our previous finding that ST10 CRISPR1-positive stains commonly carry chromosomal ermB while showing suppression of plasmid-mediated ermA (Zhang et al., 2021a), is consistent with reports by Hullahalli K et al. in Enterococcus faecalis (Hullahalli et al., 2017; Gholizadeh et al., 2021). This model suggests that CRISPR1 activity may selectively restrict the acquisition of mobile genetic elements, thereby favoring the stable vertical inheritance of chromosomal. This finding indicates that the evolutionary strategy of the ST10 lineage may involve minimizing genomic mobility and its fitness costs to protect the integrity of the core vertically inherited genome.

The convergence of high CRISPR1 prevalence, a conserved virulence gene profile, and stable chromosomal ermB resistance in ST10 points towards a model of coordinated adaptation. It is hypothesized that the CRISPR1 system contributes to the fitness of the ST10 lineage through a dual mechanism: first, by providing defense against phage and limiting genomic rearrangements, it may safeguard chromosomal loci encoding both virulence factors and the ermB gene; second, by impeding the uptake of certain plasmids, it may reduce genetic load and selectively favor clones that rely on vertically inherited, chromosomally-integrated resistance. This “high-virulence/controlled-resistance” phenotype could offer a selective advantage in a setting where erythromycin pressure exists, enabling successful colonization and invasion while maintaining genomic stability.

Several limitations of this study must be acknowledged. The sample is derived from a single center and may not fully represent the heterogeneity across China. The proposed mechanistic roles of CRISPR1 in stabilizing virulence genes and restricting plasmid uptake remain speculative, as direct functional validation through genetic manipulation (e.g., CRISPR1 knockout or interference experiments (Souza-Silva et al., 2022; Gopalakrishna et al., 2023)) was not performed. The ecological context of maternal colonization and the transition to invasive disease also requires further exploration through longitudinal studies.

Despite these limitations, this study provides novel insights into the molecular epidemiology of GBS in China. It establishes a strong association between the dominant ST10 clone and the CRISPR1 system, proposing a plausible framework for how CRISPR-mediated genome stability might co-optimize virulence and antibiotic resistance. From a clinical perspective, these finding suggests that CRISPR1 could serve as a surveillance marker potentially, aiding in monitoring the spread and informing regionally tailored prophylaxis strategies. Future research should prioritize functional studies to directly test CRISPR1’s role, expand surveillance to multiple geographic regions to track clone dissemination, and employ multi-omic approaches to unravel the regulatory networks underlying the successful adaptation of the ST10 lineage.

In summary, this study reveals that the regional dominance of Streptococcus agalactiae ST10 is associated with a distinctive molecular profile featuring a high prevalence of the CRISPR1 system, a conserved virulence arsenal, and stable chromosomal erythromycin resistance. These findings highlight the potential role of CRISPR systems in shaping bacterial pathogen adaptation and underscore the importance of integrating molecular epidemiology with mechanistic insights to understand and monitor the evolution of clinically significant bacterial clones.

## Data Availability

The original contributions presented in the study are included in the article/supplementary material. Further inquiries can be directed to the corresponding author.
